# Fast source camera identification using matching signs between query and reference fingerprints

**DOI:** 10.1007/s11042-014-1985-3

**Published:** 2014-05-22

**Authors:** Yongjian Hu, Chang-Tsun Li, Zhimao Lai

**Affiliations:** 1 School of Electronic and Information Engineering, South China University of Technology, Guangzhou, 510640 People’s Republic of China; 2 Department of Computer Science, University of Warwick, Coventry, CV4 7AL UK; 3 Border Control Command College of Guangzhou, No. 399 South Olympic Road, Tianhe District, Guangzhou City, Guangdong Province China 510663

**Keywords:** Source camera identification, Fast search algorithm, Camera fingerprint digest, Robustness, Search Priority Array (SPA)

## Abstract

Fast camera fingerprint search is an important issue for source camera identification in real-world applications. So far there has been little work done in this area. In this paper, we propose a novel fast search algorithm. We use global information derived from the relationship between the query fingerprint/digest and the reference fingerprints/digests in the database to guide fast search. This information can provide more accurate and robust clues for the selection of candidate matching database fingerprints. Because the quality of query fingerprints may degrade or vary in realistic applications, the construction of robust search clues is significant. To speed up the search process, we adopt a lookup table that is built on the separate-chaining hash table. The proposed algorithm has been tested using query images from real-world photos. Experiments demonstrate that our algorithm can well adapt to query fingerprints with different quality. It can achieve higher detection rates with lower computational cost than the traditional brute-force search algorithm and a pioneering fast search algorithm in literature.

## Background

There have been a number of forensic methods proposed for establishing the relationship and linkage between digital images/videos and the imaging devices responsible for their creation (e.g., [4, 8, 18, 22]). Lukas *et al*. [18] first proposed to use the imaging sensor pattern noise (SPN) such as the Photo-Response Non-Uniformity (PRNU) of imaging sensors as camera/camcorder fingerprint for source camera/camcorder identification. Since then, a variety of SPN-based algorithms have been presented. Some explore new ways of extracting camera fingerprints and extending fingerprint applications from source camera identification to image forgery detection (e.g., [4, 8]), while others pay attention to improving quality of fingerprints (e.g., [1, 12, 14]) and the detection effectiveness (e.g., [11, 15–17]). In the meantime, the issues relating to SPN-based camera identification are raised, for example, the robustness of SPN-based camera identification [21], the trustworthiness of device SPNs [3, 6, 18], and the feasibility and reliability of applying SPN-based identification to a sizable fingerprint database [9].

Although remarkable progress has been made in developing source camera identification for real-world applications, some areas such as fast camera identification still need further research. Anticipating future use of camera identification from sensor fingerprint by law enforcement and government, fast camera identification plays an important role in two typical scenarios: (i) for a large database of *N* reference camera fingerprints, forensic analysts may want to determine whether it contains the fingerprint of the camera that took a given query/test image; (ii) given tens of thousands of query images, forensic analysts may want to search through a small or moderate-size reference fingerprint database for the identification of source cameras of those query images and group those query images according to their source cameras. The solutions to (i) and (ii) are basically the same. Therefore we do not distinguish these two application scenarios in this paper. The search for a match between the query and the database fingerprints can be formulated as a multiple-hypothesis testing problem with a cross correlation detector applied to each fingerprint in the database. The sequential search/brute-force search is a simple and traditional method. Given a database of *N* fingerprints, the average number of search rounds for the brute-force search algorithm (BFSA) is *N*/2. Since current commercial cameras are with the resolution of several megapixels, the search process may be intractably long if a large number of correlation-based comparisons have to be made. How to accurately and efficiently match query fingerprint and reference fingerprints in the database is thus of paramount importance in this application.

Few papers have been published in this area. References [2] and [10] are two early works in literature. In [2], a tree structured vector quantization-based fast search algorithm is presented. Considering that each camera has a unique SPN fingerprint and each SPN fingerprint can be modeled as an independent and identically distributed noise signal additive to an image, this tree structured algorithm does fingerprint matching on a group rather than individual basis. Before the search starts, the reference fingerprints in the camera database are evenly divided into two groups. The sum of fingerprints of each group is viewed as a composite fingerprint and becomes a node of the binary tree. Each group is further divided into two subgroups, and the composite fingerprint of each subgroup is calculated and treated as a node at a new level of the binary tree. Such a binary division continues until no division is possible. Finally, each subgroup consists of only one reference fingerprint. Given a query fingerprint, the search starts from top to bottom by picking the node that yields the higher correlation value between the query and the composite fingerprints at each level. It is reported that the logarithmic decrease in the search complexity is achieved. However, when applying this scheme to the identification of source camera in a reference camera fingerprint database, the following problem will be raised: if a query image is not taken by any reference camera in the database (i.e., the fingerprint of the query image does not reside in the database), no matter which tree branch is chosen, the decision is wrong. One solution to this dilemma is to introduce a decision threshold to let the algorithm proceed along the node where the correlation value is greater than the threshold. But query images of various contents from different cameras or even from the same camera often produce query fingerprints with different levels of quality. This gives rise to increased correlation variances, making the thresholding solution almost infeasible in practical applications.

Another early fast search algorithm is the Approximate Rank Matching Search (ARMS) [10]. The large number of correlation computations is thought of as a major obstacle to efficient search. To overcome this problem, the ARMS takes two measures: (i) introduce fingerprint digests to reduce the complexity of cross correlation computation and (ii) select candidate matching database digests (i.e., reference digests in the database) before computing every correlation value. The latter can reduce the number of correlation computations in the search process. A digest is a compact subset of the original fingerprint that can sufficiently characterize the original fingerprint. The elements of the digest are called large-magnitude components or significant components of the fingerprint in [11] and [16]. In [10], *the fingerprint digest is explicitly defined as the subset that consists of an ordered list of the k (≪n) largest components of the n-component fingerprint.* Practically, some digest elements are probably defective pixels like hot pixels (with large positive values) or dead pixels (with large negative values) on the camera sensor. The ARMS is inspired by Spearman Rank Correlation [5]. The digest elements that contribute most to the Spearman rank correlation coefficient between the query and database digests are defined as *the most influential indices/elements*. A fast match between the query and database digests relies on the rank information derived from the most influential elements. Those elements are more likely located in the beginning of the digests, so the ARMS starts the search process from the first largest element of the query digest. Using coordinates of the current element, the ARMS looks for the digest elements at the same spatial locations on database fingerprints. The database digests with non-zero elements are thought of as potential candidate matching digests. These potential candidates are further selected by the inner-loop operations. Finally, a priority is given to the selected ones during the search. If the first search round fails (i.e., the correlation values between the query and the selected database digests are less than a predefined decision threshold), the ARMS goes to the second element of the query digest and repeats the search steps. This process proceeds until a match is found or the predefined search time limit is exceeded. Since the search clue for each search round is derived from one element of the query digest a time, we call the ARMS a local information-based fast search algorithm. Usually, local information is sensitive to noise. In [10], Goljan *et al.* only gave the results of the ARMS under the assumption that both query and reference fingerprints are good quality fingerprints. However, the quality of query fingerprints cannot be guaranteed in real-world applications due to different image sources and device-dependent properties.

In this paper, we propose a new fingerprint digest-based fast search algorithm. To better handle practical query images, we propose to use global information for guiding fast search. In general, global features are more robust than local features against noise. During experiments, we observed the following phenomenon: when comparing the elements/components at the same spatial locations from a pair of camera fingerprints (i.e., SPN signals), the pair from the same camera has more elements with matching signs (“+” or “−”) than the pair from different cameras. This phenomenon becomes more apparent with the increase of fingerprint quality. Such observation motivates us to use the number of elements with matching signs between the query fingerprint and the database fingerprints for fast search clues.

The rest of the paper is organized as follows. Section 2 analyzes the problem which we face when designing fast search algorithms and describes our initial idea of improving the search order. Section 3 introduces our fast search algorithm. We elaborate on the measurement of search priority, the lookup table and the construction of the Search Priority Array (SPA). In Section 4, we use experiments to demonstrate the performance and advantages of the proposed algorithm. In Section 5, we draw conclusions.

## Problem statement and motivation

To increase search efficiency, current SPN-based fast search algorithms focus on devising a data structure which can select the most likely matching reference fingerprints from the database without resorting to a large amount of correlation computations. The major attention of [2] and [10] is paid to the construction of this data structure. For realistic applications, however, there is another challenge, i.e., robustness. We use a simple example to explain the impact of fingerprint quality on search results. Suppose a database consists of nine reference camera fingerprints {**W**_*i*_}, *i* = 1, 2 …, 9. For a given query fingerprint **X**, we further assume that **W**_7_ corresponds to the camera which is responsible for **X**. That is, **W**_7_ is the correct matching database fingerprint. Let *ρ*_*i*_ be the cross correlation between **X** and **W**_*i*_ (*i* = 1, 2 …, 9). If a correlation value is greater than the decision threshold *t*_k_, a matching database fingerprint is found. We assume that there are two cases which will occur to the database: (a) *ρ*_*i*_ ≤ *t*_*k*_, if *i* ≠ 7; otherwise, *ρ*_*i*_ > *t*_*k*_. (b) *ρ*_*i*_ ≤ *t*_*k*_, *i* ≠ 2, 7, 9; otherwise, *ρ*_*i*_ > *t*_*k*_. In case (a), the statistical detector can readily make a correct decision as there is only one correlation value greater than the threshold. In case (b), however, the situation is more complex since there are three correlation values which exceed the threshold, even though *ρ*_2_ and *ρ*_9_ are marginally greater than *t*_k_. In this case, the search order becomes significant because it affects the output of the search. For a sequential search algorithm, its detector may choose **W**_2_ as the matching fingerprint rather than **W**_7_. A wrong decision is thus made. In fact, the poor signal quality of fingerprints often results in large variances of correlation values even for the fingerprints that come from the images taken by the same camera. We can readily make database fingerprints have high quality and be at the same quality level (e.g., the same signal-to-noise ratio (SNR)), but in real-world application scenarios, however, we can hardly do the same thing to query fingerprints as they are usually extracted from the images which come from miscellaneous sources and most probably have quite different contents. Apparently, the situation described in case (b) occurs to the database more frequently. This analysis implies that we must consider the requirement of robustness when we design fast search algorithms. This challenge was not given enough attention in previous works (e.g., [2] and [10]).

In the above example, *ρ*_2_ and *ρ*_9_ are often not as large as *ρ*_7_. This is because *ρ*_2_ and *ρ*_9_ are caused by the effect of noise. Therefore, one solution to case (b) is to increase the decision threshold from *t*_k_ to *t*_k_’, which only allows *ρ*_7_ to exceed *t*_k_’. Unfortunately, such a solution can be hardly put into use in a large camera fingerprint database or a database which is searched through with the query fingerprints of different SNRs. Making the search algorithms more intelligent seems to be a more promising solution. For example, if a search algorithm has the capability to prioritize the candidate matching database fingerprints before the correlation-based comparison (e.g., one gives preference to **W**_7_ in the above example), it can wisely avoid making wrong decisions. In other words, the search order is very important. This work makes efforts in this direction. Such intelligence is essentially a kind of robustness against the noise interference. Since the ARMS also has the capability to give preference to the candidate matching database digests in the search process, we will use it as the basis of the comparison in our simulations.

## Fast search algorithm

Throughout this paper, vectors and matrices are written in bold upright font. We may index a matrix with one-dimensional index, in which case it is assumed that the matrix has been converted to a vector, for example, by rows. For image **I**, the noise residual **X** is calculated as follows:

1 $$ \mathbf{X}=\mathbf{I}-F\left(\mathbf{I}\right)=\mathbf{I}\mathbf{K}+\boldsymbol{\Xi} $$

where **K** refers to the PRNU factor and **Ξ** is the sum of independent random noise components from the camera imaging procedure and the noise component introduced by the denoising filter *F* (e.g., [20]). **X** contains both the PRNU signal **IK** and the noise item **Ξ**, and thus can be thought of as a coarse camera fingerprint. According to the analysis in [18], the averaging operation can effectively suppress the noise item in Eq. (1). So the average of multiple difference images from the images taken by the same camera can well play the role of the reference fingerprint of the camera. The average of *M* **X** *s* from the same camera, **W**, is calculated as follows:

2 $$ \mathbf{W}=\frac{1}{M}{\displaystyle \sum_{m=1}^M{\mathbf{X}}_m}=\frac{1}{M}{\displaystyle \sum_{m=1}^M{\mathbf{I}}_m{\mathbf{K}}_m}+\frac{1}{M}{\displaystyle \sum_{m=1}^M{\boldsymbol{\Xi}}_m} $$

A better approximate version of the true camera fingerprint is expected from Eq. (2) when the image number *M* is greater than 50 [18]. In this work, we use Eq. (2) with a large *M* to estimate reference camera fingerprints. We also use the same denoising filter in Eq. (1) as in [18]. Note that some articles (e.g., [4, 10]) used the estimated PRNU factor $\widehat{\mathbf{K}}$ as camera fingerprint, but these two forms of fingerprints have no fundamental differences. The presence of **IK** in the noise residual of **I** can be interpreted as evidence that **I** was taken by an imaging sensor with **K**. In order to remove the Non-Unique Artifacts (NUAs) that exist in the imaging sensors from different cameras of the same brand or model, we use the zero-meaning (ZM) operation proposed in [4] to preprocess **X** and **W**_*i*_(*i* = 1, 2 …, *N*). The main ingredients of NUAs are caused by color interpolation and the row-wise and column-wise operations of digital imaging sensors and/or image processing circuits. To remove the NUAs on the noise residual, the ZM operation subtracts the column average from each pixel in the column and then subtracts the row average from every pixel in the row. To simplify notation, we still write **X** and **W**_*i*_ rather than ZM(**X**) and ZM(**W**_*i*_). For color images, this work separately extracts the noise residual from each color band, and then integrates them into a synthetic signal using the standard luminance formula:

3 $$ \mathbf{X}=0.299{\mathbf{X}}_r+0.587{\mathbf{X}}_g+0.114{\mathbf{X}}_b $$

where **X**_*r*_, **X**_*g*_ and **X**_*b*_ are red, green and blue components of **X**, respectively.

For each search round in this work, the decision is made based on the following normalized cross correlation *ρ*:

4 $$ \rho = corr\left(\mathbf{X},\mathbf{W}\right)=\frac{{\displaystyle \sum_{i=1}^n\left(\mathbf{X}\left[i\right]-\overline{\mathbf{X}}\right)\left(\mathbf{W}\left[i\right]-\overline{\mathbf{W}}\right)}}{\left\Vert \mathbf{X}-\overline{\mathbf{X}}\right\Vert \left\Vert \mathbf{W}-\overline{\mathbf{W}}\right\Vert } $$

where **X** and **W** denote the query fingerprint and the reference fingerprint, respectively. ‖ ⋅ ‖ is the L_2_ norm and the bar “-” refers to the mean. To simplify the discussion, we assume perfect synchronization between the two signals (i.e., no geometrical distortion of images/fingerprints). Since a two-dimensional matrix can be easily transferred to a vector by rearranging the elements, say, from left to right and from top to bottom, this work does not distinguish whether an image or its fingerprint is represented by a matrix or by a vector except specific indication, as the reference [10] did. If *ρ* is greater than the decision threshold *t*_k_, the detector decides H_1_ (the query fingerprint and the reference fingerprint are responsible for the same camera); otherwise, the detector decides H_0_ (the query fingerprint and the reference fingerprint are responsible for two different cameras). It is worth mentioning that more sophisticated detectors like the PCE (Peak to Correlation Energy ratio) [7, 8] can also be used to improve the detection rates, but such complicated detectors may extremely slow down the search process. Alternatively, these detectors can be used in the after-search validation, that is, for double-checking the results from fast search algorithms.

### The measurement of search priority

**Fig. 1:**
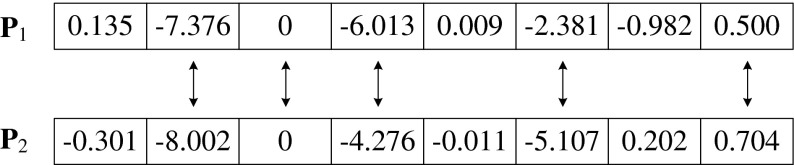
Illustration of the measurement of search priority *n*_*s*_. Here *n*_s_ = 5

This subsection elaborates on the most important concept of this work, i.e., the measurement of search priority *n*_s_. This measurement is the basis of our Search Priority Array. Assume **P**_1_ and **P**_2_ are two PRNU fingerprints. *n* = |**P**_1_| = |**P**_2_|, where | ⋅ | refers to the length of a fingerprint sequence. Let the search priority measurement *n*_*s*_ denote *the number of elements with matching signs that are located at the same spatial locations in* **P**_1_ *and* **P**_2_ (see Fig. 1). Here we count zero elements, but zero elements have no real effect on the correlation value.

To reveal the property of *n*_s_, we calculate *n*_s_ values between the two fingerprints from the same camera and from different cameras, respectively. The images come from 12 commonly used digital cameras C_i_ (*i* = 1,2…,12). Table 1 lists each camera represented by C_i_ (*i* = 1,2…,12). The correspondent camera reference fingerprint is denoted as **W**_*i*_ (*i* = 1, 2 …, 12). In this work, instead of extracting the fingerprint from the full-size image, we only crop a block of 1024 × 1024 pixels from the central part of the image and use the fingerprint extracted from this block in our experiments. This processing not only ensures the synchronization of signals but also reduces the chance of encountering saturated or distorted regions [14]. Note that the training image sets are solely used for estimating the reference camera fingerprints. To increase the quality of reference signals, each reference camera fingerprint is estimated from 100 training images using Eq. (2). On the other hand, the test image sets only act as the source of query images. Query images are never used for the estimation of reference camera fingerprints. The number of photos captured by each individual camera is listed in Table 1.

**Table 1:** Cameras used in experiments. The first 12 cameras are our own. We downloaded the photos of the rest 58 cameras from http://www.flickr.com/

No.	Model	Training set size	Test set size	No.	Model	Training set size	Test set size
C_1_	Canon PowerShot A620	100	100	C_36_	KODAK DX6490 ZOOM	100	95
C_2_	Canon IXY Digital 500	100	100	C_37_	Canon PowerShot ELPH 300 HS	100	49
C_3_	Canon Digital IXUS 850 IS	100	100	C_38_	Canon EOS 450D	100	35
C_4_	Canon PowerShot A400	100	100	C_39_	Canon EOS REBEL T3	100	29
C_5_	Canon EOS 450D	100	100	C_40_	Canon EOS REBEL T3i	100	27
C_6_	Fujifilm FinePix S602 ZOOM	100	100	C_41_	Canon 8800 F	100	25
C_7_	Panasonic DMC-LX2	100	100	C_42_	Canon PowerShot G11	100	24
C_8_	Nikon CoolPix L3	100	100	C_43_	PENTAX K-x	100	23
C_9_	Nikon D90	100	100	C_44_	Canon EOS 400D	100	19
C_10_	Olympus u820	100	100	C_45_	Leica M8	100	20
C_11_	Olympus C730UZ	100	100	C_46_	Canon EOS REBEL T2i	100	11
C_12_	Sony DSC-S40	100	100	C_47_	Olympus E-500	100	5
C_13_	Canon EOS 60D	100	149	C_48_	PENTAX K-x	100	8
C_14_	Sony DSLR-A900	100	135	C_49_	Nikon D200	100	142
C_15_	Panasonic DMC-TZ7	100	119	C_50_	Canon EOS REBEL T1i	100	59
C_16_	Olympus E-P2	100	66	C_51_	Panasonic DMC-FX100	100	199
C_17_	Canon PowerShot SD300	100	70	C_52_	Canon Digital IXUS 80 IS	100	114
C_18_	Canon PowerShot S95	100	63	C_53_	Panasonic DMC-FZ7	100	146
C_19_	Panasonic DMC-TZ3	100	59	C_54_	Casio EX-Z1080	100	22
C_20_	Samsung Digimax A7/Kenox D7	100	50	C_55_	Nikon D90	100	182
C_21_	Canon PowerShot S90	100	196	C_56_	Canon EOS 7D	100	99
C_22_	Canon PowerShot A400	100	190	C_57_	Panasonic DMC-G1	100	200
C_23_	Canon PowerShot A720 IS	100	100	C_58_	Canon PowerShot A40	100	100
C_24_	Casio EX-Z2000	100	118	C_59_	Canon PowerShot G5	100	34
C_25_	Canon EOS Kiss X3	100	104	C_60_	Canon PowerShot SD800 IS	100	5
C_26_	Canon EOS 40D	100	98	C_61_	Nikon D300	100	176
C_27_	Canon PowerShot SD1000	100	53	C_62_	Nikon E8800	100	132
C_28_	Nikon D40X	100	190	C_63_	Canon EOS 7D	100	97
C_29_	Nikon D60	100	11	C_64_	Fujifilm FinePix HS20EXR	100	229
C_30_	Canon PowerShot SX200 IS	100	172	C_65_	Nikon D3100	100	79
C_31_	Sony DSC-H70	100	58	C_66_	Canon PowerShot SD880 IS	100	99
C_32_	Nikon D90	100	200	C_67_	Canon EOS 40D	100	96
C_33_	Canon EOS 30D	100	200	C_68_	Fujifilm FinePix S4000	100	100
C_34_	Olympus E-30	100	100	C_69_	Nikon D90	100	79
C_35_	Nikon D90	100	153	C_70_	Nikon D300	100	83

**Fig. 2:**
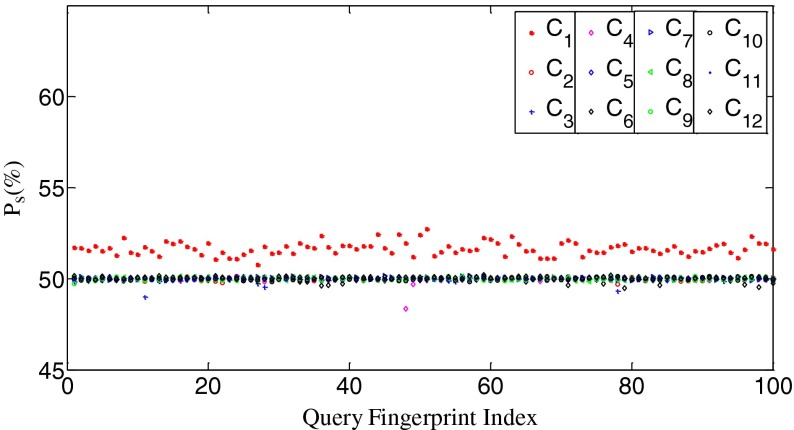
The *P*_s_ values between **W**_1_ and **X** *s* from 12 cameras, respectively. *P*_s_ = *n*_s_/*n*

We randomly choose a camera fingerprint, say **W**_1_, as the reference signal and calculate *n*_s_ values between it and query fingerprints from C_i_ (*i*=1,2...,12). The results are shown in Fig. 2. For convenience of display, we use *P*_s_ instead of *n*_s_ for the vertical axis, where *P*_s_ = *n*_s_/*n*. Figure 2 shows that the intra-class (i.e., intra-camera) *P*_s_ values scatter around 51.8 % while most of the inter-class (i.e., inter-camera) *P*_s_ values fluctuate around 50 %. The intra-class values are obviously higher than the inter-class values, and the two types of data are visibly separated.

**Fig. 3:**
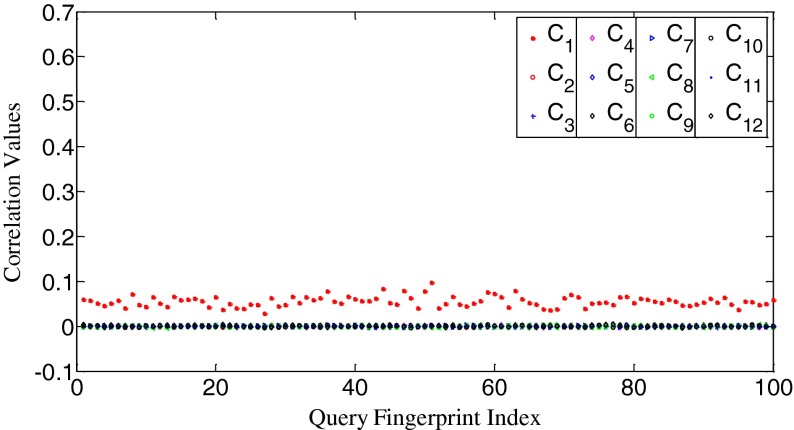
The correlation values between **W**_1_ and **X** *s* from 12 cameras, respectively

To uncover the relationship between our measurement of search priority and the correlation value, we calculate correlation values between two fingerprints from the same camera and from different cameras, respectively. The results are shown in Fig. 3. It can be seen that the intra-class correlation values scatter around 0.058 while the inter-class correlation values fluctuate around 0. Clearly, the intra-class values are higher than the inter-class values, and the two types of data are visibly separated too. By examining Figs. 2 and 3, we can easily find that *the n*_*s*_ *values and the correlation values ρ are higher for the two fingerprints from the same camera than from different cameras. Moreover, n*_*s*_ *and ρ tend to change in the same direction*.

**Fig. 4:**
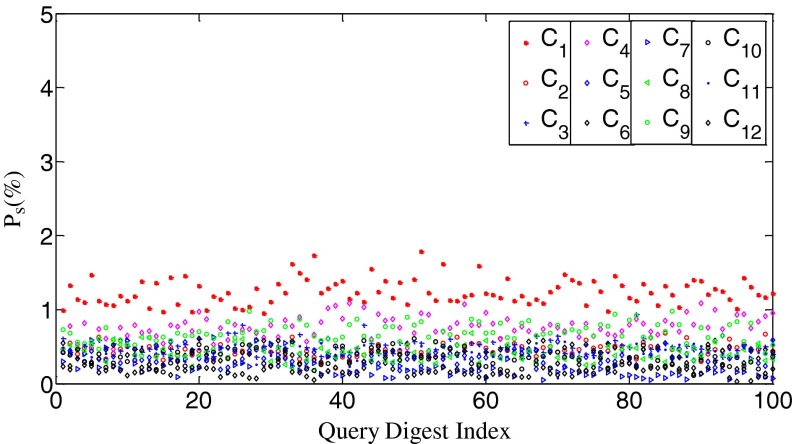
The *P*_s_ values between ${\tilde{\mathbf{W}}}_1$ and $\tilde{\mathbf{X}}s$ from 12 cameras, respectively. *k* = 10,000. *P*_s_ = *n*_s_/*k*

**Fig. 5:**
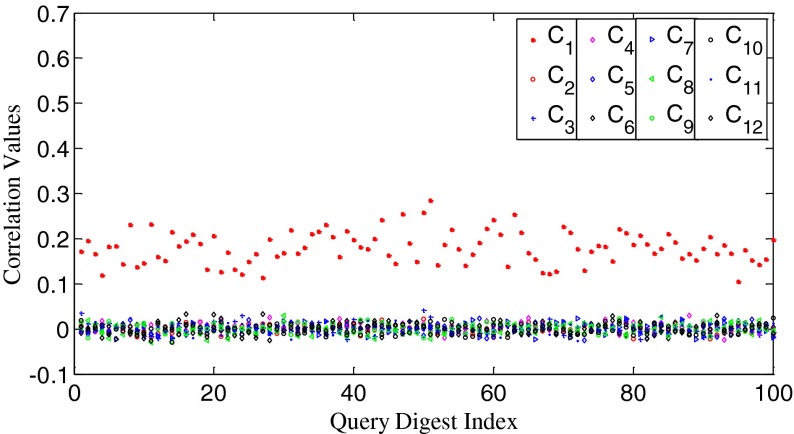
The correlation values between ${\tilde{\mathbf{W}}}_1$ and $\tilde{\mathbf{X}}s$ from 12 cameras, respectively. *k* = 10,000

Our fast search algorithm will be based on fingerprint digests, so we repeat the above experiments on digests. We set the length of digest *k* as 10,000 for the images of 1024 × 1024 pixels. Similar to [10], the elements of a fingerprint are first permuted in a descending order by magnitude, and then the first 10,000 elements constitute the fingerprint digest. The *n*_s_ values and the correlation values are shown in Figs. 4 and 5, respectively. The letters capped with “~” denote digests in this work. We can observe that the relationship between *n*_s_ and *ρ* remains unchanged. The big difference is that both the variances of the *n*_s_ values and the correlation values increase. According to our experiments, the shorter the digests are, the larger the variances. We repeat the same experiments on other test data and have observed the similar phenomena. Based on these observations, we propose our heuristic search scheme as follows: *for a given query digest, the most likely matching database digest is the one which possesses the highest n*_*s*_ *value. Therefore, the higher priority should be given to the database digest with the higher n*_*s*_ *value.* It is impossible to quantify the relationship between *n*_s_ and *ρ*. In essence, our search scheme is based on the high probability between them. That is why we cannot directly replace *ρ* with *n*_s_ during the search. We also emphasize that our discussion about the relationship between *n*_s_ and *ρ* is limited to the PRNU fingerprints of imaging devices, and we have no intention to obtain a general conclusion about the relationship between *n*_s_ and *ρ*. Since the counting of *n*_s_ involves every element within the two fingerprints, our measurement explicitly reflects the global relationship between these two signals.

### The lookup table

**Fig. 6:**
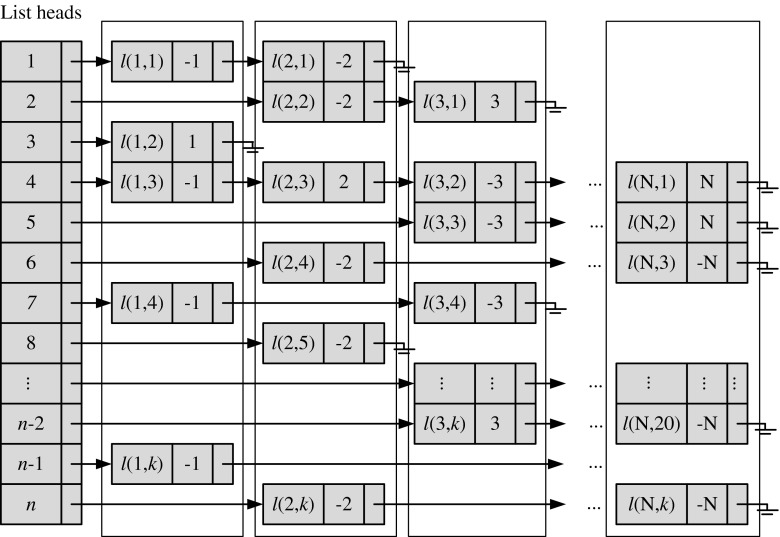
An example of the lookup table. Each column in the dash block represents a fingerprint digest

In order to determine the search priority order, we have to calculate *n*_s_ values between the given digest and every reference digest in the database. We save the resulting *N n*_s_ values in array **N**_*s*_. By sorting **N**_*s*_ in a descending order, we obtain the SPA which gives the search priority order. To accelerate the calculation of *n*_s_ between the given query digest and each database digest, this work proposes a new date structure. As shown before, when calculating *n*_s_, we do not need to know the real number of the element but its sign and location. Therefore, instead of directly visiting the database, we introduce a lookup table in which we only save necessary information of all the digests in the database, i.e., the sign and location of each digest element. Assume there are *N* reference cameras. The digest database is composed of $\left\{{\tilde{\mathbf{W}}}_i\right\}$, *i* = 1,2…,*N*. To extract the sign and location of each digest element, we define two functions as follows:

5 $$ S\left(i,d\right)=\operatorname{sgn}\left({\tilde{\mathbf{W}}}_i\left[d\right]\right)\cdot i,\kern1em 1\le i\le N,1\le d\le k $$

6 $$ l\left(i,d\right)=L\left({\tilde{\mathbf{W}}}_i\left[d\right]\right)\begin{array}{cc}\hfill, \hfill & \hfill 1\le i\le N\hfill \end{array},1\le d\le k $$

where sgn(⋅) refers to the sign function. Given a digest *i*, sgn(⋅) extracts the sign of the element located in *d* on the digest. As a result, Eq. (5) can map digest *i* into a sequence of “+*i*” and “−*i*”. *L*(⋅) makes the digest element in location *d* correspond to an element/component in its full-length fingerprint. In other words, for fingerprint *i*, *L*(⋅) returns the original coordinate of a fingerprint element which corresponds to a digest element in location *d*. More information about Eq. (6) can be found in [10]. We save the *S*(*i*, *d*) value in the linked list at entry *l*(*i*, *d*) of lookup table **H**. Hash tables are efficient structures to represent large arrays. They need less memory for storage and are easy to manage. A separate-chaining hash table is a good structure resolving hash collisions. So we use a separate-chaining hash table to build **H**. The detailed information of how to program the separate-chaining hash table can be found in [13]. An example of **H** is shown in Fig. 6. To help readers better understand its structure, we purposely show entries *l*(*i*, *d*) (*i* = 1, 2, … *N*, *d* = 1, 2, … *k*) and let column *i* correspond to digest *i*. In practical programming, we do not need to save the value of *l*(*i*, *d*). Note that " + " is omitted here. Using the separate-chaining hash table, we can efficiently calculate the *N n*_s_ values by one-layer loop with *k* × *N* operations instead of the traditional two-layer loops with *k* × *n* operations. The pseudo code of calculation of *n*_s_ is given in the next subsection.

### The search priority array

The *n*_s_ values between $\tilde{\mathbf{X}}$ and ${\tilde{\mathbf{W}}}_i\ \left(i=1,2,\dots, N\right)$ are saved in array **N**_*s*_. **N**_*s*_[*i*] is used to reflect the degree of correlation between $\tilde{\mathbf{X}}$ and ${\tilde{\mathbf{W}}}_i$. We sort the elements of **N**_*s*_ in a descending order and save the indices of the sorted elements in array **N**_*sp*_. The array **N**_*sp*_ is the so-called *Search Priority Array*. Obviously, its first element corresponds to the largest *n*_*s*_ value and it is the index of the database digest which is most likely to be correlated with $\tilde{\mathbf{X}}$. Likewise, its second element corresponds to the second most likely matching database digest, and so on. The SPA gives the search priority order with respect to a given query digest because it reflects a global relationship between the query digest and the digest database from the perspective of *n*_s_.

When a candidate database digest ${\tilde{\mathbf{W}}}_i,i\in \left[1,N\right]$ is chosen, the normalized correlation value *ρ*_*i*_ is calculated as follows:

7 $$ {\rho}_i= corr\left(\mathbf{X}\left[L\left({\tilde{\mathbf{W}}}_i\left[d\right]\right)\right],{\tilde{\mathbf{W}}}_i\right) $$

where $L\left({\tilde{\mathbf{W}}}_i\left[d\right]\right)$ (*d*=1,2...,*k*) represents the locations of elements of ${\tilde{\mathbf{W}}}_i$. In other words, we only choose the elements of **X** at $L\left({\tilde{\mathbf{W}}}_i\left[d\right]\right)$ for the correlation computation [10]. For simplicity, we will use $L\left({\tilde{\mathbf{W}}}_i\right)$ in the rest of the paper.

**Fig. 7:**
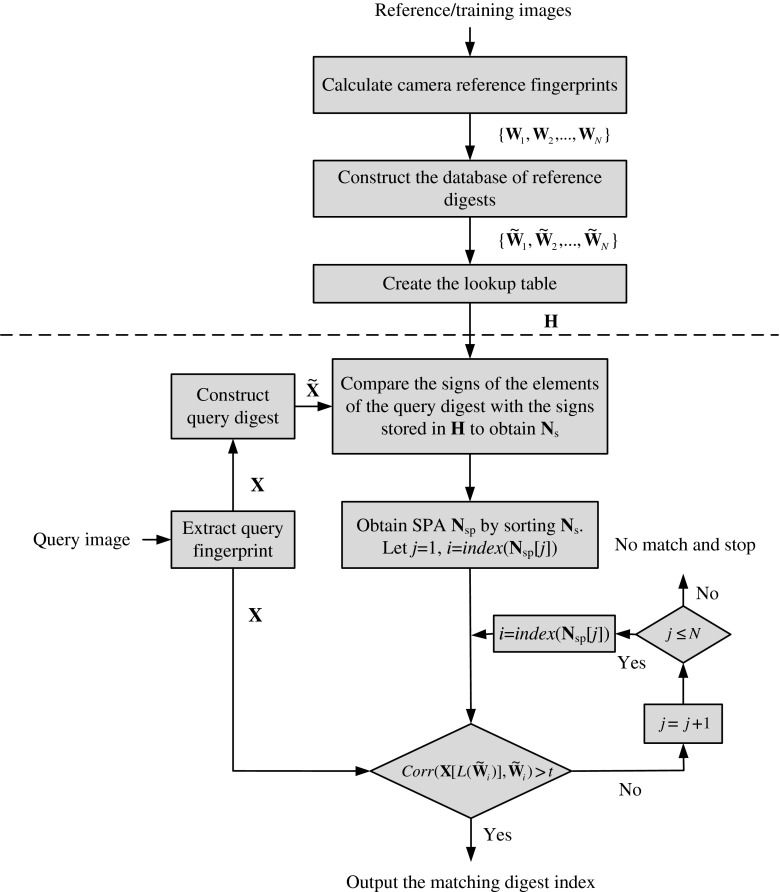
The flowchart of our fast search algorithm. The operations above the dash line belong to the offline phase while the operations below the dash line belong to the online phase

The following pseudo codes describe the implementation of the above procedures in details. The complete flowchart of the proposed fast search algorithm can be found in Fig. 7. For convenience of discussion, we divide our search algorithm into two phases: offline and online. Steps 1–8 below constitute the offline phase, which consists of the calculation of database fingerprints/digests, the construction of the digest database and the construction of the lookup table. Steps 9–27 constitute the online phase, which consists of the calculation of query digest, the construction of the SPA, and the search loop.

% Calculate the full-length database fingerprints and then their correspondent digests
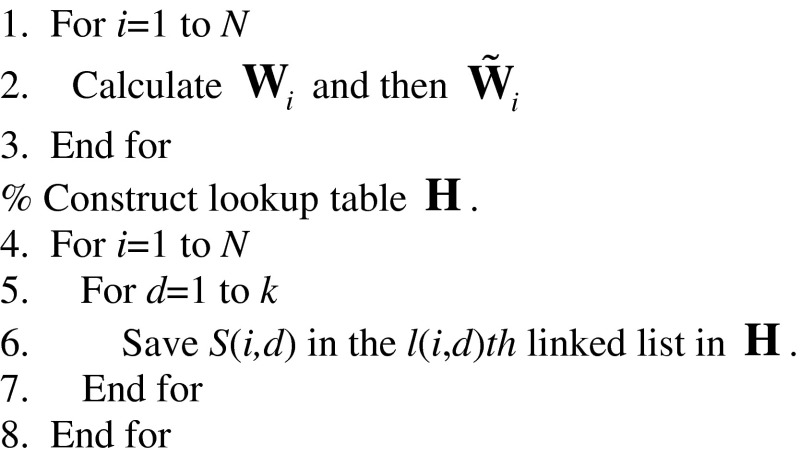


% For a given query digest, calculate **N**_*s*_. Sort its elements in a descending order and save the indices of the elements in **N**_*sp*_ to obtain the SPA.
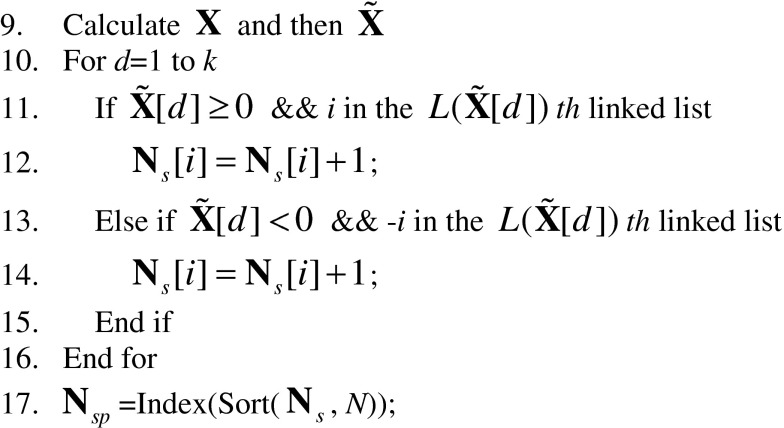


% Carry out the search based on the search priority order determined by the SPA.
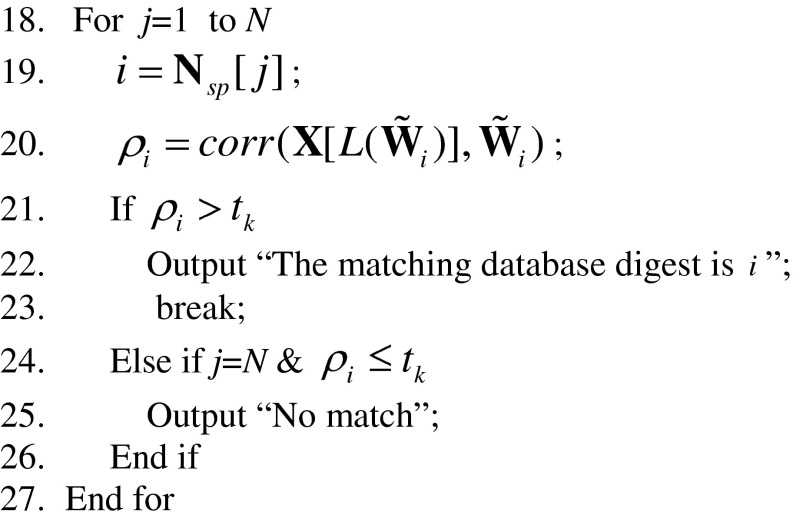


## Experiments and discussions

The ROC (receiver operating characteristic) analysis provides an unbiased description of algorithm performance without suffering from the arbitrary selection of a decision threshold [19]. So the ROC curves are used to describe the overall performance of our algorithm. We also evaluate its performance by the missed detection rate (equivalent to the false negative rate) and the false positive rate (equivalent to the false alarm rate). The false positive rate is defined as [No. False Positive decisions]/[No. actually negative cases] while the missed detection rate is defined as 1-[No. True Positive decisions]/[No. actually positive cases] [19]. In addition, we give the average number of search rounds, and the average search time at significant decision thresholds. To demonstrate the advantages of our algorithm, we compare it with the early fast search algorithm ARMS and the traditional sequential search algorithm BFSA. The authors of [10] gave two sets of parameters for the ARMS: Mode A with parameters *w* = 1,000 and *t*_cand_ = $\sqrt{w}$ =31.623, and Mode B with *w* = 1,000 and *t*_cand_ = 0.2 $\sqrt{w}$ = 6.3246. The parameter *w* is used to control the window of the outer-layer/outer search loop. The parameter *t*_cand_ is the threshold for the accumulated evidence, which is introduced to control the inner-layer/inner search loop. When the digest length *k* = 10,000, the time for performing one cross correlation computation on our computer is about 1.8 × 10^−4^ s. Hence the upper limit of search time for the ARMS is set to 1.8 × 10^−4^ × 2 × (*N*/2). The reader is referred to [10] for more detailed information about the ARMS.

Our simulations involve 13,696 images captured by 70 cameras, as shown in Table 1. The first 60 cameras (i.e., C_1_-C_60_) constitute the reference camera database. To evaluate the missed detection rate and the false positive rate, we use query fingerprints that come from all the 70 cameras (i.e., C_1_-C_70_). In order to simulate a more challenging environment, we intentionally include some images which come from the same camera model or make but from different people. For example, we have five Nikon D90 models in the database. As stated before, C_1_-C_12_ are our own cameras. And the images captured by our own cameras are with the native resolution. Most of those images are scenic photos captured under daylight. On the other hand, the images taken by C_13_-C_70_ are downloaded from a public website http://www.flickr.com/. We have no control over the resolution, content, quality and sources of the downloaded images. All the images are saved in JPEG format. Our simulations are conducted on the platform of an Intel i5-2410 M CPU, 2.30GHz with 4G RAM. All the three search algorithms are implemented using Visual C++ 2008.

### Performance evaluation - scenario I

**Fig. 8:**
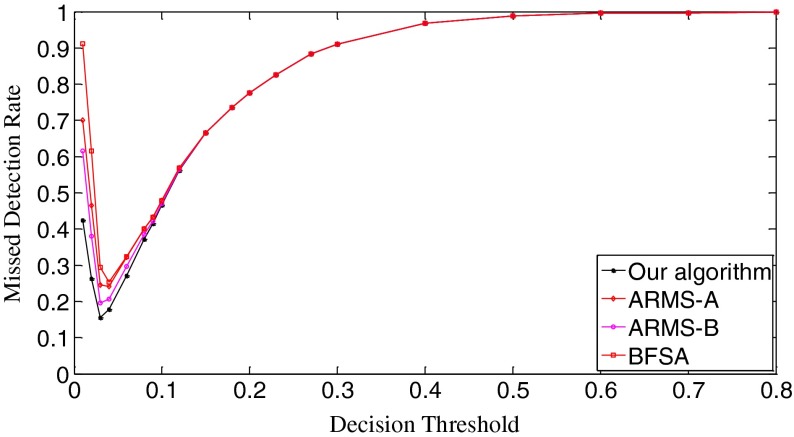
The decision threshold versus missed detection rate curves of the three algorithms. *k* = 10,000

**Fig. 9:**
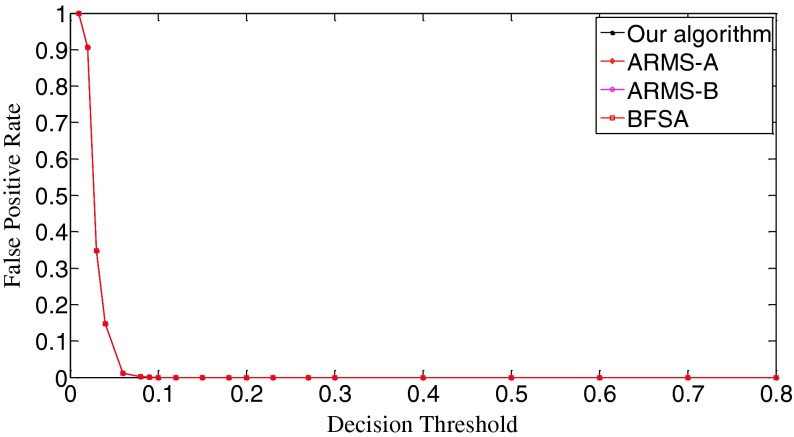
The decision threshold versus false positive rate curves of the three algorithms. *k* = 10,000

In Scenario I, each query fingerprint is extracted from one test image using Eq. (1). Such query fingerprints are of very poor signal quality due to the effects of image content (e.g., edge and texture), image storage format (e.g., JPEG compression and with JPEG compression factors), and denoising filtering. In total, we have 6,696 query fingerprints. The digest length *k* is set as 10,000, about 1 % of the full-length fingerprint (1024 × 1024 pixels). In Fig. 8, we give the decision threshold versus the missed detection rate curves. The missed detection rate reflects the capability of an algorithm to detect query images whose camera fingerprint digests indeed reside in the database. When the decision threshold *t*_k_ is 0.01, the missed detections are serious. The BFSA has the highest missed detection rate, reaching about 0.91; at the same time, our algorithm has the lowest rate, which is about 0.42. Such high rates of missed detections are mainly caused by noise interference. For example, the NUAs from the imaging sensors of the same camera brand or model might not be completely removed from query fingerprints, resulting in a high proportion of correlation values exceeding this low decision threshold. With a small increase of decision thresholds, however, the detection results of all three algorithms become much better. When *t*_k_ = 0.03, the missed detection rates of our algorithm and the ARSM-B drop to the lowest points, 0.155 for our algorithm, and 0.196 for ARMS-B. Whereas, the ARMS-A and BFSA respectively touch their bottoms of the missed detection rates at *t*_k_ = 0.04, 0.242 for ARMS-A and 0.254 for BFSA. Here ARMS-A and ARMS-B correspond to the ARMS with Mode A and Mode B, respectively. So our algorithm has the lowest bottom among the four decision threshold versus missed detection rate curves. After reaching the valley bottoms, the missed detection rates of all three algorithms climb with the increase of decision thresholds. The explanation for this phenomenon is that even for the query and the database digests from the same camera, some noise components, in particular, those caused by image content and denoising filtering, weaken their correlations. From *t*_k_ = 0.15, all three algorithms have the same missed detection rate. When *t*_k_ = 0.8, the missed detection rates exceed 0.999 for all three algorithms. Figure 9 gives the decision threshold versus the false positive rate curves. The false positive rate describes how a search algorithm reacts to query images whose fingerprint digests do not reside in the database. It can be observed that all curves are overlapped. So all three algorithms have the same false positive rates under the same thresholds, meaning that this type of error is not caused by the search schemes employed but results from the setting of decision thresholds. Looking at Figs. 8 and 9, we can find that the lowest missed detection rates and the lowest false positive rates cannot be obtained simultaneously. Interestingly, for many practical applications, it is not necessary to have a low false alarm rate because more sophisticated detectors can be run as a double check on all the fingerprints identified as candidates by the search. Too many candidate fingerprints, however, would slow down the search [10]. We call the threshold at which the decision threshold versus the missed detection rate curve reaches its bottom the significant threshold. For different algorithms, the significant thresholds may be different. The selection of good decision thresholds for fast search algorithms is a complex problem. Further discussions are beyond the scope of this paper. The reader is referred to related references (e.g., [10]) for more information.

**Fig. 10:**
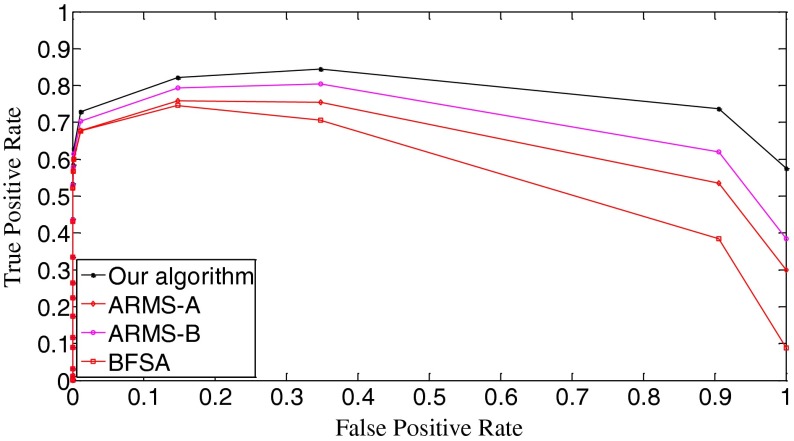
The ROC curves of the three algorithms. *k* = 10,000

In Fig. 10, we draw the ROC curves of the three algorithms. The true positive rate is equal to [No. True Positive decisions]/[No. actually positive cases]. Better decision or detection performance is indicated by an ROC curve that is higher and to the left in the ROC space [19]. Apparently, our algorithm outperforms the other two algorithms. Figure 10 also demonstrates that the ARMS is very sensitive to the setting of operational parameters because the curves of the ARMS-A and the ARMS-B have obvious differences. The setting of those parameters is related to the reference digest database. And the optimization of those parameters requires prior knowledge of the database. Goljan *et al*. gave detailed analysis of their effects and showed how to set them in [10]. In general, those parameters are mutually dependent, making it difficult to find a good combination. This is an apparent drawback of the ARMS.

**Fig. 11:**
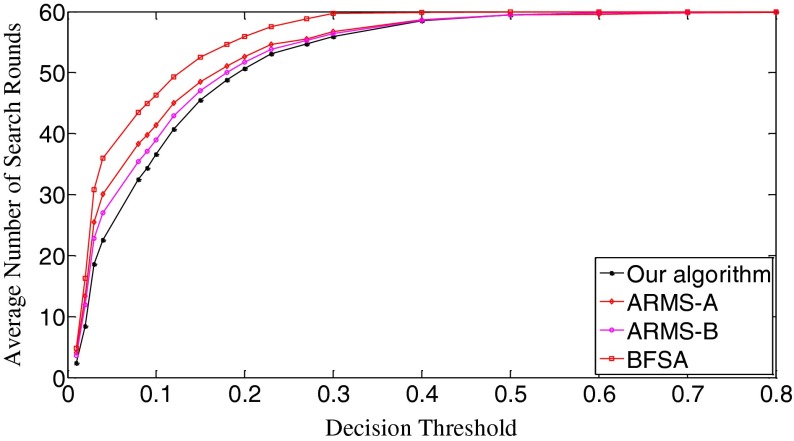
The decision threshold versus average number of search rounds curves of the three algorithms. *k* = 10,000

In Fig. 11, we can observe that the average number of search rounds is proportional to decision thresholds. Our algorithm requires the least search rounds before all three algorithms reach the same value. The BFSA does not employ any *apriori* knowledge and only carries out a sequential search, so it needs more search rounds than the others. The ARMS makes use of the approximate ranking information of the influential elements, which reduces the number of search rounds. But such local information is not as robust against the artifacts from poor quality query digests as the global information provided by the SPA of our algorithm. This explains why our algorithm requires even less search rounds than the ARMS. It is worth mentioning that our algorithm requires impressively less search rounds at those significant thresholds (i.e., 0.03 and 0.04) where the missed detection rates of the three algorithms are at their bottoms, respectively. Table 2 gives the average search time of the three algorithms at these significant thresholds. Our algorithm runs fastest among the three algorithms, which verifies that our algorithm is also the most efficient algorithm to deal with poor quality query images. Note that, in either Table 2 or Tables 3 and 4 which will appear in the next two subsections, the search time for our algorithm covers all the online operations like the generation of query fingerprint digest, the SPA construction as well as the digest comparison.

**Table 2:** The average search time at significant thresholds

Algorithm *t*_k_	Our algorithm	ARMS-A	ARMS-B	BFSA
0.03	6.833	14.066	9.588	8.013
0.04	8.220	14.766	10.491	9.581

*k* = 10,000. Time unit: milliseconds

**Table 3:** The average search time at significant thresholds for query digests with better signal quality

Algorithm *t*_k_	Our algorithm	ARMS-A	ARMS-B	BFSA
0.04	5.737	11.121	6.525	4.859
0.06	6.488	11.719	7.141	8.908

*k* = 10,000. Time unit: milliseconds

**Table 4:** The average search time at significant thresholds

Algorithm *t*_k_	Our algorithm	ARMS-A	ARMS-B	BFSA
0.03	41.471	22.366	16.635	37.872
0.04	43.829	24.755	18.083	42.947

*k* = 50,000. Time unit: milliseconds

### Performance evaluation - scenario II

**Fig. 12:**
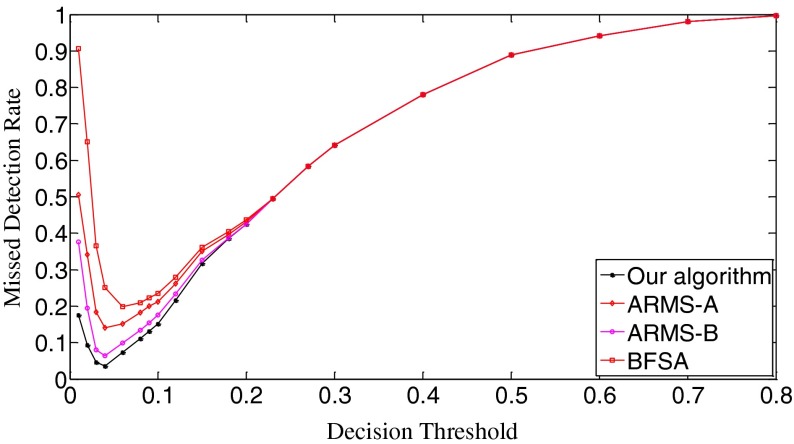
The decision threshold versus missed detection rate curves of the three algorithms. *k* = 10,000

**Fig. 13:**
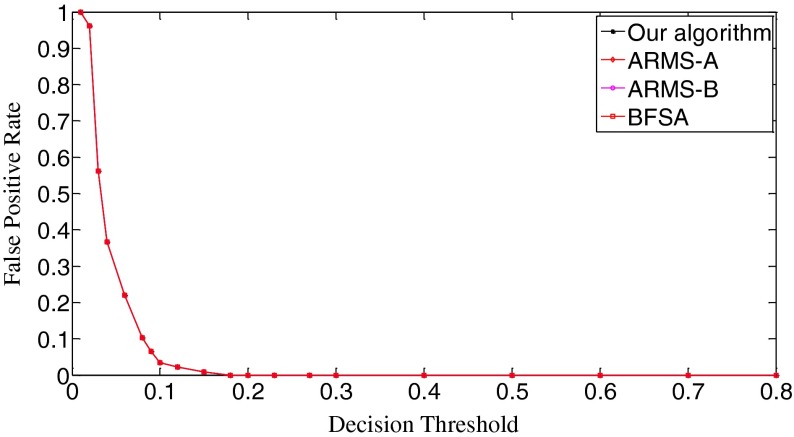
The decision threshold versus false positive rate curves of the three algorithms. *k* = 10,000

**Fig. 14:**
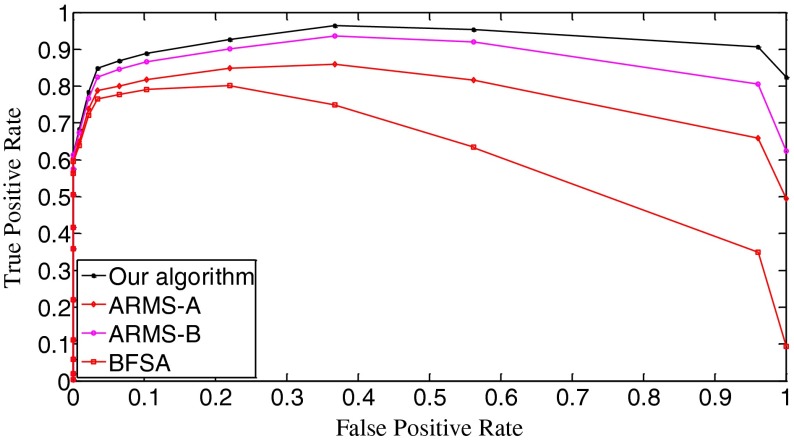
The ROC curves of the three algorithms. *k* = 10,000

In Scenario II, we evaluate the performance of our search algorithm using a little better quality query fingerprints and examine the trend of performance change. In particular, we use five test images from the same camera instead of one and average their image noise residuals in a manner of Eq. (2). Such an average value is used as a query fingerprint. In this way, 1,316 query fingerprints from 70 cameras are obtained. Figure 12 shows the missed detection rates of the three algorithms. Evidently, the behavior of all three algorithms becomes better. The initial missed detection rates of all the algorithms except the BFSA are greatly lower than their counterparts in Fig. 8. As for the valley bottoms, our algorithm has the missed detection rate as low as 0.035 (*t*_k_ = 0.04) while the ARMS has 0.141 (*t*_k_ = 0.04) for Mode-A and 0.064 (*t*_k_ = 0.04) for Mode-B, respectively. The BFSA has the highest value, i.e., 0.198 (*t*_k_ = 0.06). After passing the valley bottoms, the missed detection rates climb with the increase of decision thresholds. But compared with the curves in Fig. 8, these curves have much smoother slopes. From *t*_k_ = 0.23, the missed detection rates of the three algorithms tend to be the same. As for the false positive rates, Fig. 13 shows that all three algorithms have identical performance. In comparison with the curves in Fig. 9, the sharpness of these curves is reduced. We examine the thresholds that correspond to the bottoms of the missed detection rates and find that the false positive rates in Scenario I and Scenario II change little for all three algorithms. This joint information of the missed detection rates and the false positive rates demonstrates that the search algorithms achieve better results with the improved quality of query digests. In fact, by comparing Fig. 14 with Fig. 10, we can easily see that all the ROC curves move higher and further to the left in the ROC space, meaning that all the three algorithms perform better. Still the ROC curve of our algorithm is the highest, indicating that our algorithm retains the best overall performance among the three algorithms.

**Fig. 15:**
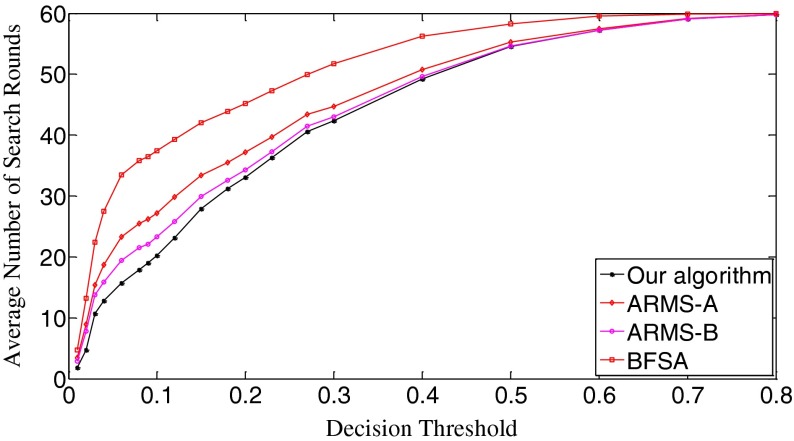
The decision threshold versus average number of search rounds curves of the three algorithms. k=10,000

Figure 15 exhibits the average number of search rounds. The slopes of all the curves become much smoother than their counterparts in Fig. 11, meaning the matching between query digests and database digests often requires less search rounds for the same threshold. When *t*_k_ < 0.6, the curve of our algorithm is the lowest, showing that our algorithm has the best accuracy in selecting the candidate matching database digests. Table 3 gives the average search time of the three algorithms at significant thresholds. Our algorithm runs faster than the ARMS. But it runs a little slower than the BFSA at *t*_k_ = 0.04. This is attributed to the fact that erroneous detections make the BFSA break its search loop earlier than normal. In fact, the missed detection rate of our algorithm is lower than its counterpart of the BFSA by 0.216 at *t*_k_ = 0.04 (see Fig. 12). According to the results in this subsection, we can easily find that the quality of query images can greatly affect the performance of fast search algorithms. Our algorithm has the best flexibility among the three algorithms.

### Performance evaluation - scenario III

In Scenario III, we investigate the effect of the digest length on the performance of the proposed algorithm. We extend *k* from 10,000 to 50,000 and use the same query fingerprints as those in Section 4.B. Hence, the number of fingerprints is still 1,316. When *k* = 50,000, the time for performing one cross correlation computation on our computer is longer than 1.8 × 10^−4^ s. Normally, the upper limit of search time for the ARMS should be increased. For simplicity of comparison with the results in the last two subsections, however, we do not change it.

**Fig. 16:**
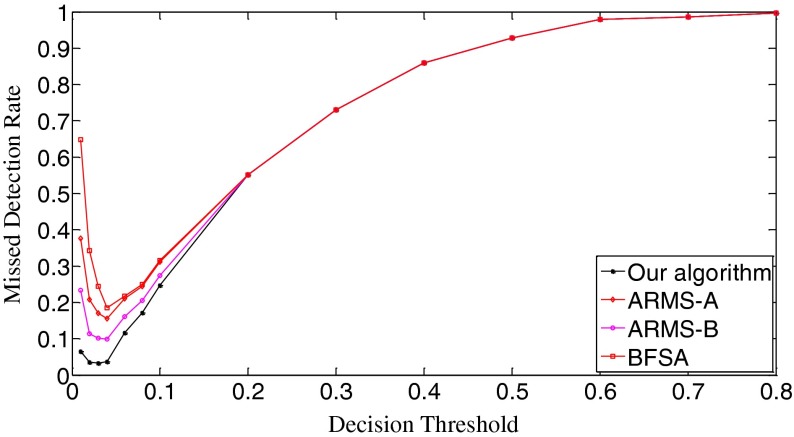
The decision threshold versus missed detection rate curves of the three algorithms. *k* = 50,000

**Fig. 17:**
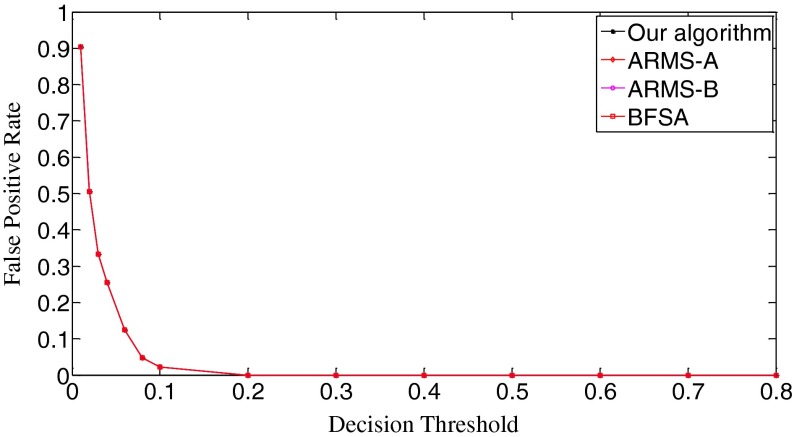
The decision threshold versus false positive rate curves of the three algorithms. *k* = 50,000

**Fig. 18:**
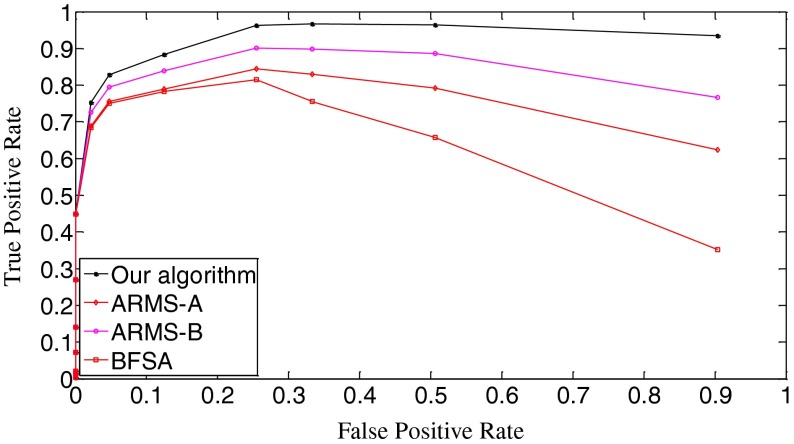
The ROC curves of the three algorithms. *k* = 50,000

**Fig. 19:**
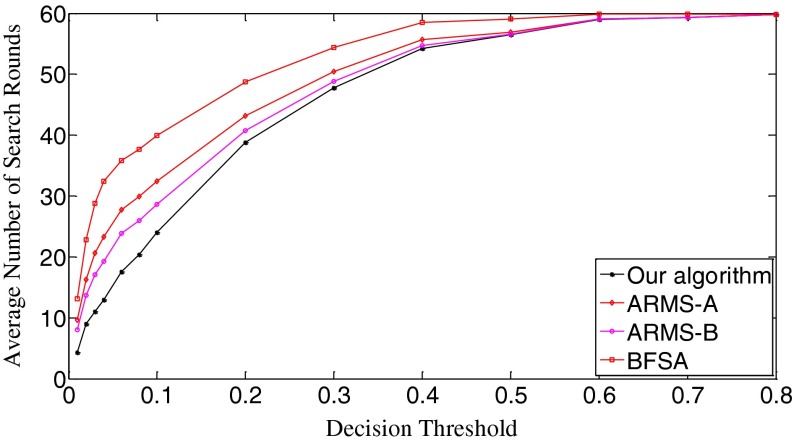
The decision threshold versus average number of search rounds curves of the three algorithms. *k* = 50,000

In Fig. 16, the initial missed detection rates of all three algorithms are further lowered compared to those in Fig. 12, but such an improvement is greater for the BFSA than for our algorithm and the ARMS. When investigating the valleys of the curves, we find that both our algorithm and the BFSA only slightly lower their bottoms, 0.033 (*t*_k_ = 0.03) for our algorithm and 0.185 (*t*_k_ = 0.04) for the BFSA; whereas the ARMS surprisingly has slightly higher bottoms, 0.156 (*t*_k_ = 0.04) for ARMS-A and 0.098 (*t*_k_ = 0.04) for ARMS-B. After passing the bottoms, the curves even climb faster with the increase of thresholds than those in Fig. 12. With respect to the false positive rates, Fig. 17 shows that the curves move moderately lower and further to the left compared with Fig. 13. But in Fig. 18, the ROC curves of all three algorithms are not as high in the left space as those in Fig. 14, which indicates that the overall performance of all three algorithms degrades. When looking at Fig. 19, we can observe that the curves of all three algorithms become a little steeper than their counterparts in Fig. 15, meaning more search rounds are often required for the same thresholds before reaching the stable value. Table 4 shows that all three algorithms require extraordinarily longer search time.

The use of long digests can reduce the variances of both *n*_s_ and the correlation. This conclusion can be easily inferred by comparing Fig. 2 with Fig. 4 and Fig. 3 with Fig. 5. This seemingly implies that we could improve the performance of fast search algorithms by increasing the length of digests. According to the results in this subsection, however, the benefits of using longer digests are only limited to the missed detection rates for very small decision thresholds, say, *t*_k_ < 0.03. Taking the increased computational cost into consideration, we now find that the use of long digests is not a recommendable approach. Specifically, for our algorithm, long digests result in a large separate-chaining hash table, and thus require more time on the calculation of *n*_s_ and the construction of the SPA. These computational costs greatly harm the search speed of our algorithm, as shown in Table 4. On the other hand, because the ARMS is based on local information, the increase of digest length does not have much positive impact on its performance. As for the BFSA, the good effect of using longer digests is also limited to the performance for very small thresholds.

### Comparison of computational complexity

The computational complexity of the three algorithms at significant decision thresholds has been compared using the average search time in Section 4.1– 4.3 In this subsection, we briefly compare their practical search manipulations. For the BFSA, its offline process includes Step 1 to Step 3 of the pseudo codes of our algorithm in Section 3.3. If the digest of a query image resides in the reference digest database, the average online computational load is *N*/2 times of computing Eq. (7); otherwise, the online computational load is *N* times of computing Eq. (7). Here we assume that there is no erroneous detection; otherwise, the BFSA may terminate its search process earlier than normal. We also assume that the time for memory access and comparison operations is trivial compared with the time for correlation computation.

For the ARMS, the first three steps of the offline process are also the same as ours. The ARMS then builds a sparse *n* × *k* matrix **S** that plays a role similar to our lookup table **H**. The elements of **S** are the database digest indices, *i* ∈ {1, 2 …, *N*}. In the online phase, the ARMS selects the candidate matching database digests based on the most influential elements. Potentially, every element of the query digest can generate a round of search. Note that such a search round corresponds to the outer loop in the ARMS. The average number of search rounds is equivalent to the average number of iterations in the outer loop in this paper. Each outer loop includes an inner loop which is used to further determine the candidate matching digests before performing Eq. (7). As mentioned before, the parameter *w* corresponds to the size of search window of the outer loop. The inner-loop parameter *t*_cand_ controls whether to carry out the correlation computation. A lower *t*_cand_ allows more correlation computations. Not only the number of iterations in the outer loop but also the number of iterations in the inner loop relies on the quality of digests. Therefore, the number of search rounds is dynamic. If the quality of query digest is high, the matching database digest would be found in the first few search rounds; otherwise, more search rounds are required. In the worst case, the ARMS requires more search rounds than the BFSA. The authors of [10] switched the ARMS to the BFSA to avoid this situation. The reader is referred to [10] for more information.

As for our algorithm, it builds hash table **H** in the offline phase. Since a sparse matrix is usually realized with a hash table, the offline computational load of our algorithm is similar to that of the ARMS. In the online phase, however, our algorithm needs to construct the SPA, which includes the calculation of the *N* elements of **N**_*s*_ and the sorting of **N**_*s*_. Although the calculation of *N n*_s_ is not complex and only involves comparisons of signs and addition operations, the sorting operation is often time-consuming for a large array. Due to this online cost, our algorithm may run slower if the digest is too long (e.g., in Section 4.3). For short digests, however, our fast search scheme is very efficient, as shown in Section 4.1 and 4.2.

## Conclusion

In this paper, we have proposed a simple and effective fingerprint search algorithm for fast source camera identification in real-world applications. Considering the quality problem of practical query fingerprints, we have proposed to improve the fast search algorithm by enhancing the robustness. Previous algorithms did not pay enough attention to this aspect. The major contribution of this work is the introduction of the Search Priority Array. The robustness of the Search Priority Array is based on the global information derived from the relationship between the query digest and all the reference digests in the database. Another contribution is that we introduce the separate-chaining hash table as the look-up table which can facilitate the construction of the Search Priority Array. As shown in experiments, our algorithm can better adapt to query images in practical applications. Experimental results have demonstrated obvious improvement over the ARMS in [10] and the BFSA in terms of the correct detection rates and the computational complexity at significant thresholds. Another advantage over the ARMS is that our algorithm does not rely on any operational parameters except the threshold, which make it behave consistently. In future, we will focus on how to extract a more accurate fingerprint and construct the digest which can better reflect the characteristics of the fingerprint. Such an effort can further reduce the computational complexity of our fast fingerprint search algorithm.
